# Approaching active matter from biophysics perspective

**DOI:** 10.2142/biophysico.bppb-v22.0020

**Published:** 2025-08-30

**Authors:** Masayuki Hayakawa, Ibuki Kawamata

**Affiliations:** 1 Department of Mechanical Engineering, Kyoto Institute of Technology, Kyoto 606-8585, Japan; 2 Division of Physics and Astronomy, Graduate School of Science, Kyoto University, Kyoto 606-8502, Japan

## Introduction

Collective motion, in which individual units move in a coordinated manner, is ubiquitous in self-propelled systems that span both biological and non-biological realms and has become one of the central themes in biophysics. As fascinating biological examples, the striking coherence of bird flocks and fish schools has long captivated scientists, while the intricate dynamics of living cells observed under the microscope continue to inspire scientific inquiry. Such systems, composed of self-propelled units, are known as active matter. A hallmark of active matter systems is their nonequilibrium nature: each constituent continuously consumes energy, sustaining persistent motion. This nonequilibrium state enables globally ordered collective motion to emerge spontaneously, even when the underlying local interaction rules are remarkably simple.

Theoretical research in statistical physics has made a huge progress in the study of collective motion in active matter systems. A significant milestone in theoretical research was the introduction of the Vicsek model, which describes the collective motion in a system of self‑propelled particles [1]. In the model, each particle follows a minimal rule set, aligning its direction of motion with that of its neighbors. The Vicsek model revealed that collective motion, a form of macroscopic order, can spontaneously emerge from simple local interactions under nonequilibrium conditions. Subsequent studies showed that various aspects of collective motions observed in both biological and synthetic systems can be captured by the Vicsek model and its extensions.

Along with the theoretical research, recent improvements in microscopy, manipulation, and micro- and nano-fabrication have enabled the controlled construction and quantitative analysis of a wide range of active matter experimental systems. In the course of these developments, the concept of active matter has broadened; it now tends to encompass any self-propelled entity that continually consumes energy and remains out of equilibrium, regardless of whether it forms ordered collective motion. This broader perspective has positive consequences. Especially, within the Biophysical Society of Japan, which attracts researchers from diverse fields, this shared focus on “self-propulsion” is drawing scientists toward active matter research.

To highlight recent advances in active matter research within the biophysical community, we are organizing a symposium entitled “Approaching Active Matter from Biophysics Perspective” at the 63rd Annual Meeting of the Biophysical Society of Japan, to be held in Nara, Japan, from 24 to 26 September 2025. Here, we invite seven early-career researchers from diverse fields who are developing cutting-edge experimental systems including cells, microorganisms, droplets, molecular motors, colloids, and so on. This symposium aims to shed new light on themes distinctive to the Biophysical Society of Japan—for instance, the origin of life and the fundamental nature of autonomy and complexity—through in-depth discussions of active matter.

## Symposium talks on synthetic active matter

Several talks in this symposium highlight synthetic active matter systems, many of which are constructed using biomolecules and chemical reactions. Since around 2010, research on building such non-living systems with life-like behavior has been actively developed, particularly within the community of the Biophysical Society of Japan [2]. These systems, though non-living, display spontaneous behaviors such as directional motion, oscillation, collective movement, and shape transformation. By leveraging reaction-driven dynamics, the speakers have begun to engineer minimal active matter systems that increasingly exhibit dynamic and intelligent forms of activeness. This progress opens up new possibilities for exploring nonequilibrium physics and for constructing life-like systems from the bottom up.

Controlling and understanding the behavior of molecular-scale active matter is an essential step toward realizing functional molecular robots. One promising approach involves the use of programmable DNA chemical reaction networks to direct the motion of molecular components. In this context, Dr. Ibuki Kawamata, the first speaker in this symposium, constructs systems in which microtubules, driven by kinesin motors on a glass surface, act as active matter. Their movement is regulated by DNA interactions designed using DNA computing technology, allowing precise and flexible control [3]. His presentation introduces an approach that links DNA reactions with active matter to program autonomous, time-evolving dynamics at the nanoscale.

The second speaker, Dr. Hirotake Udono presents recent progress in realizing life-like motion in synthetic cells constructed from DNA-based condensates. His research focuses on DNA droplets, which are liquid-state assemblies formed from nanostructured DNA motifs. These droplets exhibit directional motion when exposed to constant visible light. This photo-responsiveness is achieved by incorporating azobenzene into the DNA motifs, allowing continuous light-driven behavior without the need for UV–Vis switching. Under polarized light, the droplets undergo strong elongation, reminiscent of cell division. Dr. Udono demonstrates how molecular design combined with light stimuli can generate life-like behavior in synthetic systems in his talk.

Recent advances in the design of artificial nanomotors using DNA nanoparticles are the focus of the presentation by Dr. Takanori Harashima. The DNA nanoparticle motor, known as the fastest artificial nanomotor, exhibits two-dimensional super-diffusive motion via a burnt-bridge Brownian ratchet mechanism driven by enzymatic RNA hydrolysis [4]. However, achieving unidirectional motion on a two-dimensional surface remains challenging because lateral motion cannot be suppressed. In this presentation, Dr. Harashima introduces design principles that address this limitation by introducing anisotropy into the motor or its rail. His work offers new insights into the control of nanoscale dynamics in synthetic active matter systems.

The design of synthetic molecular systems that exhibit life-like behavior through self-sustained chemical oscillations is the subject of Dr. Muneyuki Matsuo’s presentation. These synthetic systems are designed to sense, move, and function by linking chemical reactions to changes in their physical properties. A central feature is a recursive mechanism: the reaction alters the system’s physical properties, which in turn affect the reaction rate. This feedback loop brings the system back to its original state, enabling continuous, autonomous oscillations. The presentation highlights how such design principles can give rise to emergent, life-like dynamics in artificial molecular systems.

## Symposium talks on biological active matter

Biological active matter systems also receive attention in this symposium, with several talks focusing on living organisms. These studies, where biological function and physical dynamics are tightly coupled, represent the very essence of biophysics. The systems under investigation range from bacteria and motile algae to social amoebae, and their collective behaviors help illuminate fundamental principles of organization in nonequilibrium systems. Unlike studies on synthetic active matter, the focus here is more on understanding natural phenomena than on constructing systems, often through physical approaches. Such investigations inspire new directions in pure statistical physics and shed light on the physical principles underlying morphogenesis and collective behavior.

How gravity affects the behavior of motile microorganisms such as *Paramecium*, *Tetrahymena*, and *Chlamydomonas*—all of which exhibit gravitaxis, a directional response to gravity—is the focus of a presentation by Dr. Azusa Kage, the fifth speaker. While gravitational biology has often focused on larger organisms, the presentation in this symposium highlights the gravity-dependent behavior and collective motion of these unicellular systems [5–8]. Dr. Kage discusses how such phenomena can be framed within the context of active matter. The talk also explores future directions for gravitational biology involving microorganisms, including the integration of deep learning-based image analysis techniques [9] to enhance the understanding of microorganism behavior under varying gravitational conditions.

The sixth speaker, Dr. Kazusa Beppu, introduces a novel strategy for controlling collective dynamics in a suspension of *Bacillus subtilis* by combining ferrofluids with external magnetic fields [10]. This approach utilizes magnetic torque in a magnetizable medium to align non-magnetic swimmers, providing a versatile method to modulate their collective behavior. By applying magnetic fields in different orientations, a variety of dynamic states can be induced and systematically explored. Continuum modeling and Lattice-Boltzmann simulations offer insights into the mechanisms behind these states. His presentation demonstrates the potential of magnetic control as a powerful tool for investigating instability-driven phenomena in active matter systems.

A mutant strain of *Dictyostelium discoideum* cells serves as the subject of a presentation by Dr. Masayuki Hayakawa. In this unusual yet informative system, the cells are unable to show chemotaxis. Remarkably, even though these cells cannot respond to such external cues, they exhibit spatiotemporal patterns and collective motion through self-organization [11,12]. This raises fascinating questions about the mechanisms underlying spontaneous collective behavior, and provides a valuable model for bridging biological organization and the physics of active matter. The presentation is expected to offer new insights into how complex behavior can emerge from minimal rules, contributing to our understanding of both morphogenesis and nonequilibrium systems.

## Summary

To summarize, this symposium will serve as a platform to explore the frontiers of active matter research from a biophysical perspective, covering both synthetic and biological systems. We believe that the presentations and discussions held here will not only deepen our understanding of active matter studies, but also inspire future developments in biophysics, soft matter, and bioengineering.
